# Characterizing Variations in Binding Kinetics of Affinity Biosensors: A Boundary-Layer and Site-Quality Approach

**DOI:** 10.1021/acsomega.6c07361

**Published:** 2026-09-14

**Authors:** Brandon Tipper, Lisa M. Miller, Steven Johnson, Mohammad Nasr Esfahani

**Affiliations:** † School of Physics, Engineering and Technology, 8748 University of York, York YO10 5DD, U.K.; ‡ York Biomedical Research Institute, University of York, York YO10 5DD, U.K.

## Abstract

Affinity biosensors rely on biological recognition elements immobilized on a transducer surface to capture specific target analytes. Binding kinetics between target molecules and surface-immobilized receptors are commonly analyzed using the Langmuir interaction model, which assumes homogeneous binding sites with constant kinetic parameters. Here, we present a computational framework representing the evolution of apparent binding kinetics using a normalized coverage-dependent distribution function. The model assumes that binding at relatively low surface coverage is dominated by more accessible and favorably oriented receptor sites, followed by increasing contributions from heterogeneous binding environments as surface coverage increases. The kinetic formulation was coupled with fluid-flow and convection–diffusion modeling to account for mass transport and near-surface residual analyte concentration. The framework was evaluated using quartz crystal microbalance with dissipation measurements of IgG and C-reactive protein antibody–antigen interactions under varying experimental conditions. Quantitative assessment using normalized root-mean-square error (NRMSE) showed consistently improved agreement with the experimental measurements relative to the classical Langmuir model, with one benchmark comparison yielding an NRMSE below 1.5% for the proposed model compared with more than 4% for the classical model. The results demonstrate that incorporating coverage-dependent apparent kinetics improves the description of the investigated high-affinity binding responses and provides a phenomenological framework for characterizing heterogeneous surface binding. By providing a more detailed description of coverage-dependent binding behavior, the proposed framework has the potential to support the design and optimization of affinity biosensors under practical operating conditions.

## Graphical abstract

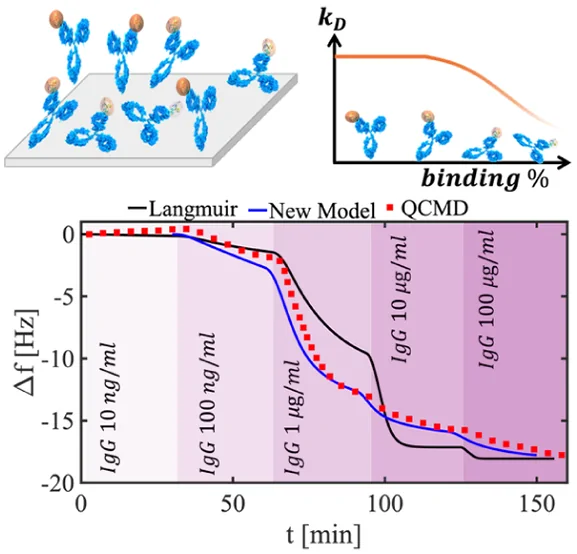

## 1 Introduction

Affinity biosensors have unique capabilities for rapid, sensitive, and specific detection of biomolecules, such as proteins, nucleic acids, and small metabolites, with a wide range of applications across medical diagnostics, environmental monitoring, and biomedical research.^1^ The molecular selectivity of these analytical devices is provided by recognition elements that are immobilized on a transducer surface and that interact with a target analyte with high specificity and affinity. Many of these techniques, including surface plasmon resonance (SPR),^2^ bio-layer interferometry (BLI),^3^ and quartz crystal microbalance with dissipation (QCMD),^4^ can perform time-resolved measurements of these interactions. This allows quantification of binding kinetics that are essential to determine their performance, as a higher rate of association between a target analyte and a surface-immobilized recognition element increases the slope of the response curve, leading to a faster response time as well as an improved limit of detection.^5^ Quantifying binding kinetics is also critical in drug discovery where both the association rate and duration of association determine drug efficacy.^6^ Despite their importance, accurate determination of binding kinetics is technically challenging, as the measurements are highly sensitive to assay design, sample preparation, and data analysis. For example, a global benchmark study of the kinetics of high affinity interactions revealed errors of over 60% in the derived association and dissociation rates due to differences in assay preparation and execution.^7^ The primary source of these errors is the sensitivity of kinetic rate measurements to experimental protocols. In addition to experimental protocols, the models used to describe the kinetics of molecular interactions can introduce further systematic errors in the estimated rate constants and subsequent sensor design.

The Langmuir isotherm, derived from statistical mechanics, is widely used for modeling kinetic measurements in biosensors.^4^ This model assumes a uniform distribution of target analytes in the solution-phase and that all receptors have equivalent energy levels with no interactions between receptors resulting in consistent binding kinetics. These simplified assumptions fail to accurately reflect the nature of molecular interactions in practical biosensor technologies. For instance, the variation in local density of immobilized recognition elements, the vibration of a QCMD sensor (slowing ligand binding)^8^ and the charge fluctuation in an SPR chip^9^ result in heterogenous binding kinetics due to inhomogeneities in the local environment.^10^ Several recent experimental studies have further supported the notion that binding kinetics can change over time, such as sensor aging causing more nonspecific binding over time,^11^ and functionalisation of the surface leading to a randomness in the orientation of the recognition elements and their inter-molecular interactions.^12^ The variation of binding kinetics becomes more significant in low affinity interactions, where the environment of interaction has impact on both association rates and equilibrium state.^13^

The limitations of the Langmuir isotherm in describing experimentally observed biosensor responses have motivated the development of alternative models that account for heterogeneous binding kinetics.^14^ Fractal analysis, for example, describes adsorption and diffusion on heterogeneous surfaces, allowing apparent binding kinetics to evolve over time.^15^ Other approaches have characterized binding as a distribution of interactions rather than a single kinetic process. Forssén et al. developed the Adaptive Interaction Distribution Algorithm (AIDA), which derives continuous rate constant distributions from measurements at multiple analyte concentrations, demonstrating that assuming a single interaction can lead to significant fitting errors.^16,17^ Similarly, Svitel et al. used multi-concentration assays to identify multiple affinity populations and visualized binding behavior as a continuous kinetic landscape.^18^ Vanjur et al. further showed that a single dissociation constant is insufficient to characterize DNA interactions on sensor surfaces, as changes in surface chemistry and electrostatic repulsion can distort the apparent binding kinetics.^19^ While these studies provide evidence for heterogeneous and concentration-dependent binding behavior, they generally rely on fixed interaction classes or static parameter distributions and therefore do not fully capture the continuous evolution of local surface environments and their influence on binding kinetics during an experiment.

To address this limitation, we propose an alternative framework for quantifying the evolution of binding kinetics arising from changing surface characteristics during a sensing experiment. The approach introduces a normalized kinetic distribution that describes binding interactions as a continuous function of surface state rather than a fixed set of parameters. This distribution is integrated with mass transport modeling based on the Navier–Stokes equations, enabling the simulation of microfluidic biosensors while simultaneously tracking the evolution of binding kinetics throughout the reaction. The proposed method was evaluated against experimental measurements on IgG antibody-antigen interactions detected by QCMD technology. Unlike conventional methods that represent heterogeneous interactions using discrete affinity classes or static parameter distributions, the proposed framework explicitly captures the continuous shift in apparent binding behavior as surface coverage increases. The method can be applied to individual experiments or used to investigate trends under varying operating conditions, such as changes in flow rate or analyte concentration, with model performance evaluated quantitatively using NRMSE and changes in binding behavior characterized through parameters such as the initial association rate and distribution variance. As a result, the framework provides a useful approach for analyzing dynamic binding environments and identifying coverage-dependent transitions from approximately homogeneous to heterogeneous apparent binding kinetics.

In the remainder, theoretical models are presented first by discussing the development of algorithm and boundary conditions for a biosensor integrated with a microfluidic channel. This is followed by a discussion of experimental measurements, based on QCMD analysis of antibody-antigen interactions and which are used to assess the novel analytical models. This work concludes with a discussion on the importance of considering variation in binding kinetics and informing design considerations for affinity biosensors.

## 2 Method

### 2.1 Computational Method

The biosensor system can be considered as a fluidic channel (commonly a microfluidic channel) which contains a sample matrix that is exposed to the functionalized surface of the biosensor transducer. A general 2D model can be considered for such a system, as shown in Figure 1. Target biomolecules flow through convection and diffusion from the inlet of the system to the outlet, diffusing to the sensor-sample interface causing binding between target biomolecules in the sample matrix and receptors immobilized on the sensor surface. In this work, two approaches will be considered in a reaction limited regime; (i) a uniform distribution of receptors with constant binding kinetics (known as the Langmuir isotherm) (Figure 1 a), (ii) non-uniform distribution of receptors with a continuous variation in binding kinetics, proposed in this study (Figure 1 b).

**Figure 1:**
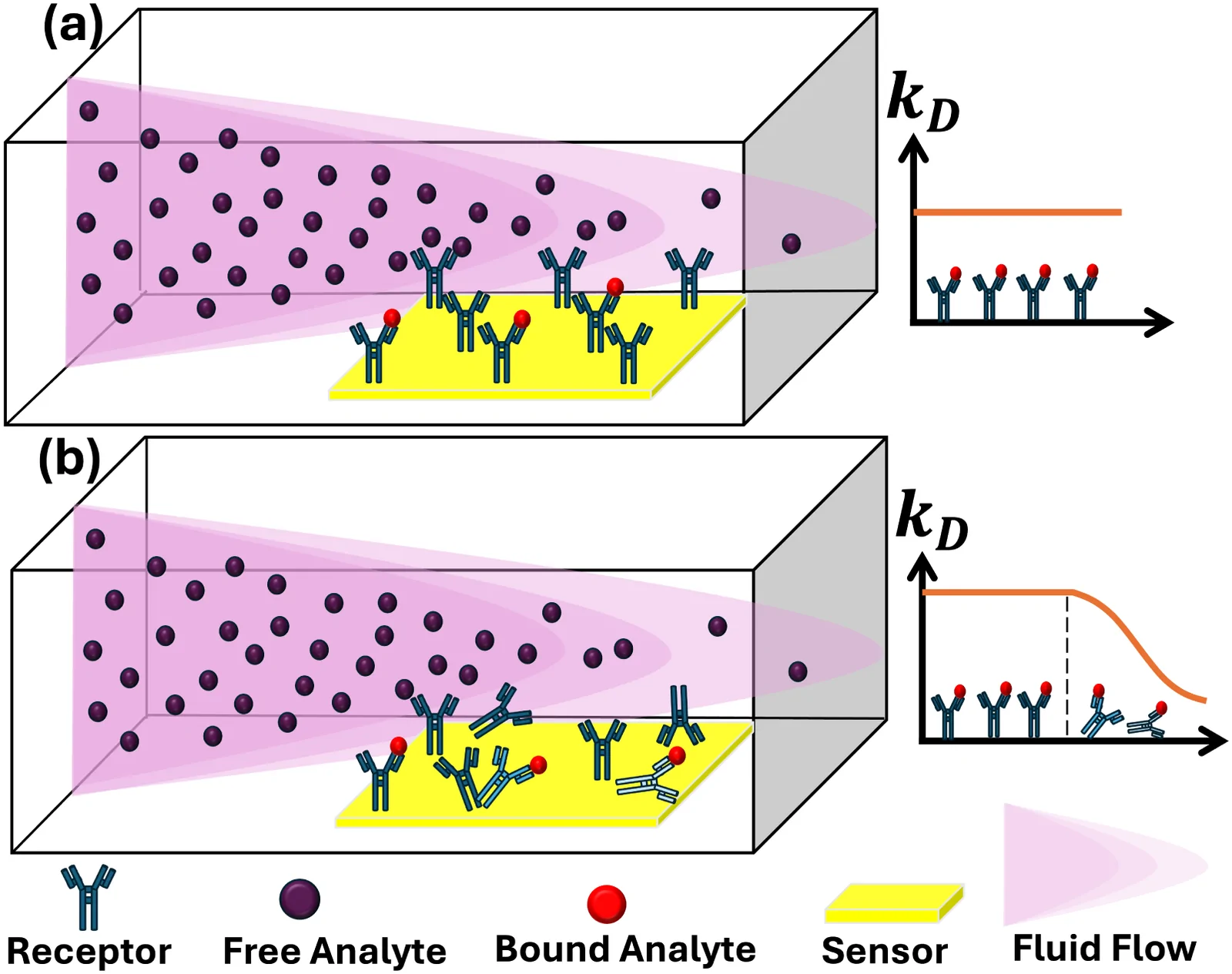
Kinetic model of a functionalized surface integrated with a microfluidic channel, representing affinity biosensors. In this model, input analyte flows in a parabolic profile on the left, running over the sensor’s surface and undergoing binding with immobilized receptors described by two models (a) the classical Langmuir isotherm that assumes a uniform distribution of surface-immobilized receptors with constant binding kinetics, independent of the fraction of bound receptors and (b) the proposed approach which models a non-uniform distribution of receptors with binding kinetics that change with respect to the percentage of bound sites.

The incompressible form of the Navier–Stokes equation can be used to model fluid and analyte flow. First, theoretical calculations will be presented to model the microfluidic integrated biosensor depicted in Figure 1. Convection and diffusion together can be modeled using the Navier–Stokes equations, which originate from the Cauchy momentum model. In an incompressible 2D fluid flow (*x*, *y*), the Navier–Stokes equations can be expressed as:

1 $$\frac{du}{dx}+\frac{dv}{dy}=0$$

2 $$\frac{du}{dt}+u\frac{du}{dx}+v\frac{du}{dy}=-\frac{1}{\rho }\frac{dp}{dx}+\mu \left(\frac{d^{2}u}{dx^{2}}+\frac{d^{2}u}{dy^{2}}\right)$$

3 $$\frac{dv}{dt}+u\frac{dv}{dx}+v\frac{dv}{dy}=-\frac{1}{\rho }\frac{dp}{dy}+\mu \left(\frac{d^{2}v}{dx^{2}}+\frac{d^{2}v}{dy^{2}}\right)$$

where *ρ* is the density of the medium (*kg m* ^–3^), *μ* is viscosity of the medium (*kg m* ^–1^ *s* ^–1^), *p* is the pressure (*kg m* ^–1^ *s* ^–2^), *u* is the velocity (*ms* ^–1^) in the *x*-direction and *v* is the velocity in the *y*-direction (*ms* ^–1^). By considering the Fick’s first law as:

4 $$J=-D\frac{dc}{dx}$$

where *J* is the diffusive flux (*Mm* ^–2^ *s* ^–1^), *D* is the diffusion constant (*m* ^2^ *s* ^–1^), *c* is concentration (*Mm* ^–3^) and *x* is the position (*m*), with the fluid flow from the Navier–Stokes equations, the way in which concentration flows through the system can be modeled using the following equation:

5 $$\frac{dc}{dt}+Û\times \nabla c=\nabla \times \left(D\nabla c\right)+S$$

where *S* is the sink/source term. It is important to note that this system has two possible modes of mass transport-limited or reaction-limited, depending on the system characteristics. This can be determined by the Péclet and Damköhler numbers.^20^ It should be noted that all experiments used in this paper are reaction-limited. In affinity biosensors, the Langmuir isotherm is widely used to model the binding interactions between receptors and target molecules. The general first-order Langmuir isotherm can be expressed as:

6 $$\frac{d\theta }{dt}=k_{on}C_{0}\left(1-\theta \right)-k_{off}\theta$$

where *θ* is the fraction of occupied binding sites, *C*_0_ is the analyte concentration, *k*_*on*_ is the association rate, *k*_*off*_ is the dissociation rate. The affinity of the interaction is determined mainly based on the equilibrium dissociation constant, *k*_*D*_ as $k_{D}=\frac{k_{off}}{k_{on}}$ . To determine the binding kinetic parameters from measurements, one needs to incorporate the Langmuir isotherm with the mass transfer models (eqs 1 [Disp-formula eq5]) to find the best fit to the sensor response. There are two main assumptions in this model: 1) the binding kinetic parameters are constant and homogeneous across the layer of immobilized affinity recognition elements, and 2) following dissociation, the target molecules immediately diffuse from the functionalized surface into the bulk flow. These assumptions can impose a significant discrepancy between the model and experimental observations. Here we propose a new solution to address these two assumptions by introducing a novel nonlinear algorithm that accounts for variation in the binding kinetics.

In the proposed approach, a boundary layer is assumed at the surface of the sensor, formed from target molecules that have dissociated from the functionalized surface to form a localized concentration. This means that dissociated molecules can re-bind to receptors without immediately diffusing back to the system, which is a common theory considered in previous studies.^21^ To quantify the contribution of near-surface boundary-layer effects independently from that of heterogeneous binding kinetics, both the classical Langmuir and proposed models were evaluated with and without the boundary-layer treatment using identical experimental datasets. The corresponding NRMSE analysis (Supporting Information, Section S1.2 and Figure S1) demonstrates that inclusion of the boundary layer substantially improves the classical Langmuir prediction, whereas its incremental effect is smaller when incorporated into the proposed model. This comparison indicates that both transport/re-binding and heterogeneous site quality contribute to the measured response, although the coverage-dependent kinetic description accounts for a substantial proportion of the deviation from the classical Langmuir behavior.

In addition, we also introduce heterogeneity in the kinetics of the analyte-receptor interaction, allowing the binding kinetics to change as a function of binding complex, which changes with respect to time. Interactions between target molecules and exposed binding sites on the functionalized surface are influenced by the density and orientation of the immobilized affinity recognition elements,^22^ among other factors. This in turn, leads to variation in the binding interactions and binding kinetics, depending on the approach used to functionalize the sensor surface. In the proposed model, two kinetic regimes are considered as a function of surface coverage, *θ̂*. where the maximum coverage is *θ̂*_*max*_. At relatively low coverage, binding is assumed to be dominated by receptor sites that are favorably oriented and sterically accessible. In this regime, the ensemble-averaged apparent kinetic parameters are approximated as constant values, $k_{on}^{*}$ and $k_{off}^{*}$ . As surface coverage increases, these favorable binding sites become progressively occupied and receptor sites with lower accessibility or less favorable local environments contribute increasingly to the measured response. The fitted critical coverage, *θ̂*_*c*_, defines the transition between the early, approximately constant-kinetics regime and the later, coverage-dependent heterogeneous regime. Thus, *θ̂*_*c*_ should be interpreted as an effective transition parameter rather than a directly measured fraction of a specific microscopic receptor population. For *θ* > *θ̂*_*c*_, the variation in apparent binding kinetics is represented by the normalized distribution function Φ­(*θ̂*), as

7 $$\Phi =\int_{0}^{100-{\mathrm{\theta ̂}}_{c}}\left[\frac{2\alpha }{\beta }-\frac{\alpha^{2}}{\beta^{2}}\right]d\mathrm{\theta ̂}$$

Here *α* = *θ̂* – *θ̂*_*c*_ represents the level of heterogeneous binding in percentage and $\beta =\frac{100\%\;+\;{\mathrm{\theta ̂}}_{c}}{2}$ defined as is the modal average of the binding kinetics in the heterogenous complexes. The binding kinetic constants change as a coefficient of such normalized Gaussian distribution, Φ, where fitting to experimental measurements can be used to determine homogeneous kinetic parameters, i.e., $k_{on}^{*}$ and $k_{off}^{*}$ , level of homogeneous binding, *θ̂*_*c*_, and variation of kinetics as a function of binding complex, Φ. Having biosensor response as a function of time for a range of analyte concentrations, one can implement the proposed method to obtain the variation of binding kinetics between a target molecule and immobilized receptors based on the flowchart shown in Figure 2. At first, the classical Langmuir method is applied to the sensor response at each analyte concentration to estimate initial kinetic parameters as a function of binding level, where the range of homogeneous binding and variation of kinetics in eq 7 can be determined. Using those initial parameters, Navier–Stokes eqs (eqs 1–5) can be incorporated with the Langmuir isotherm (eq 6), where the kinetic is determined at each time-step based on the level of binding complex. Then the model prediction can be compared with experimental measurements to find the difference and error. The distribution function and constant parameters then are adjusted accordingly to reduce the error. Details of the numerical solution, domain discretization and boundary conditions can be found in the (Supporting Information Section S1).

**Figure 2:**
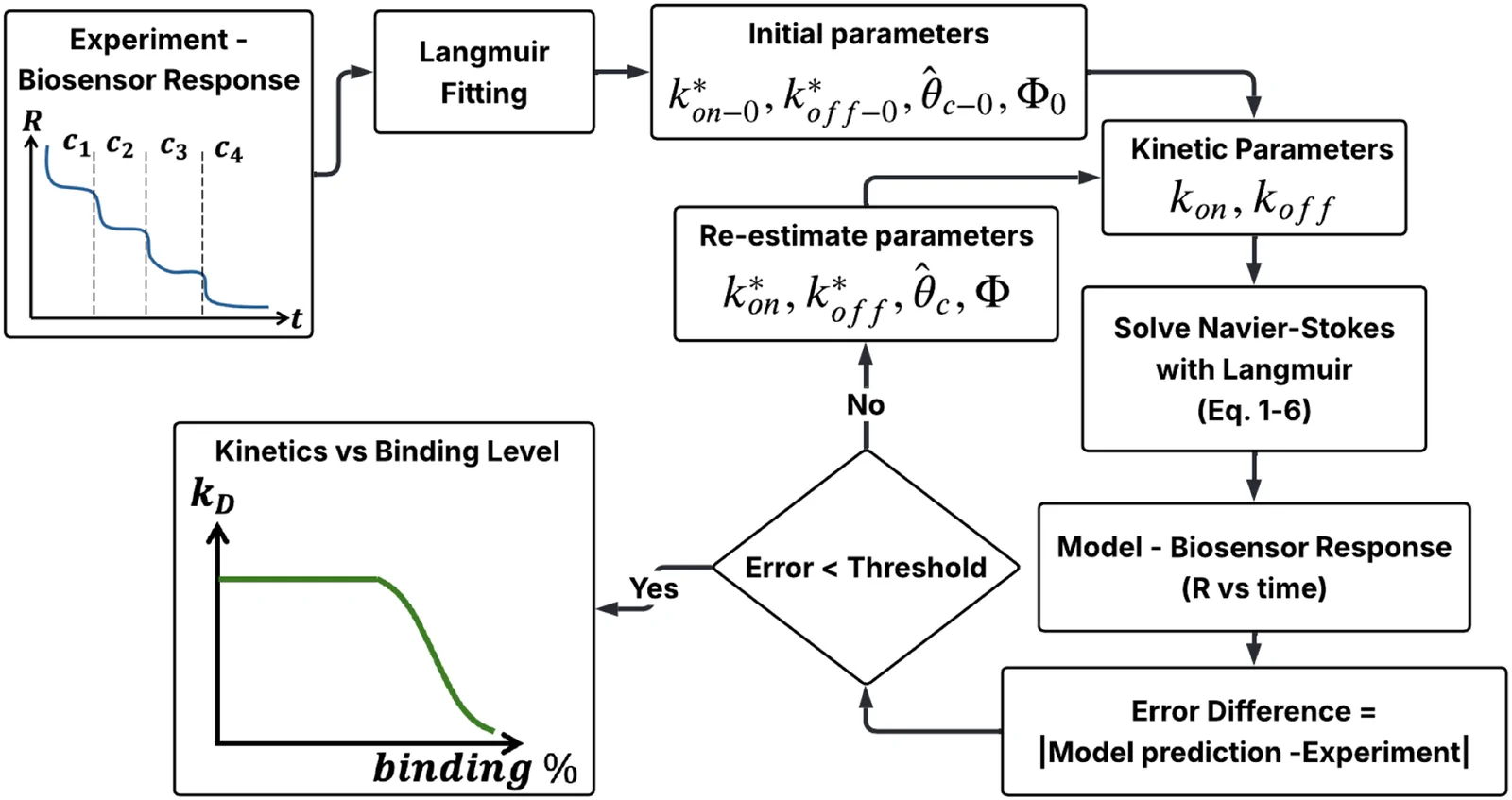
Flowchart framework to implement the proposed model, with input as experimental measurements of biosensor response, *R*, as a function of time for different analyte concentrations (e.g., *c*_1_ – *c*_4_), where kinetic function can be developed based on iterative approach to find $k_{on}^{*}$ , $k_{off}^{*}$ , *θ̂_c_* and Φ for the best fit between model and experiments.

Discrepancies between the model and experimental measurements can be used to guide the adjustment of fitted parameters. For example, if the gradient of the initial response lies within the prescribed tolerance, the estimated $k_{on}^{*}$ may be considered acceptable. However, if equilibrium is reached too rapidly, the late-stage binding behavior is not being captured correctly. In this case, *θ̂*_*c*_ should be reduced so that the system leaves the constant *k*_*D*_ regime at a lower surface coverage, thereby slowing the approach to equilibrium and improving agreement with the later portion of the experimental curve. Conversely, if the equilibrium response is within tolerance but the initial binding rate is low, $k_{on}^{*}$ should be increased to improve the early-time fit. Because increasing $k_{on}^{*}$ may otherwise lead to an overestimation of the final response, compensatory changes may also be required in the estimated saturation level or in (*θ̂*_*c*_), which controls the transition from constant to variable binding kinetics. These examples illustrate that the fitted parameters are interdependent. A parameter that lies within its individual tolerance may still need to be adjusted when a closely related parameter produces significant error elsewhere in the response curve. Through a few iterations a threshold for the difference between the model prediction and experiment can be achieved, where the kinetic parameters as a function of binding level will be the output of the model.

Here modeling attempts were made using Φ as a crucial component, and through implementing the flow chart demonstrated in Figure 2, where the following function was found to be effective to model the corresponding distribution of binding kinetics for IgG antibody-antigen interactions measured by QCMD sensors:

8 $$k_{on}=\{\begin{array}{ll} k_{on}^{*} & \mathrm{\theta ̂}\leq {\mathrm{\theta ̂}}_{c}.\\ k_{on}^{*}\Phi^{k\alpha } & \mathrm{\theta ̂}>{\mathrm{\theta ̂}}_{c}. \end{array}$$

9 $$k_{off}=\{\begin{array}{ll} k_{off}^{*} & \mathrm{\theta ̂}\leq {\mathrm{\theta ̂}}_{c}.\\ k_{off}^{*}\Phi^{k\beta } & \mathrm{\theta ̂}>{\mathrm{\theta ̂}}_{c}. \end{array}$$

This normalized model is defined here as a statistical approach to the complex system of binding kinetics, incorporating all influential factors into a single function by taking advantage of the high number of binding events to effectively resolve the probability into a given rate function. The parameters $k_{on}^{*}$ and $k_{off}^{*}$ therefore represent the apparent association and dissociation rates associated with the initial approximately homogeneous kinetic regime, whereas *θ̂*_*c*_ determines the coverage at which coverage-dependent heterogeneity becomes significant. The powers applied to Φ in eqs 8 and 9 determine the sensitivity of the apparent association and dissociation rates to this increasing heterogeneity. These powers are empirical shape factors rather than theoretically derived molecular constants. Values of *kα* = 2 and *kβ* = 2.5 were found to describe the anti-IgG/IgG QCMD measurements considered here, allowing the association and dissociation rates to evolve differently with surface coverage and consequently producing a coverage-dependent effective *k*_*D*_.

The model therefore assumes that the experimentally observed binding response represents an ensemble of continuously varying binding environments, with more favorable interactions contributing preferentially at low surface coverage and less favorable interaction contributing increasingly as coverage rises. Determination of these effective kinetic parameters requires measurements over multiple analyte concentrations. The model is phenomenological and does not uniquely identify microscopic receptor populations or molecular energy distributions; consequently, *θ̂*_*c*_, the distribution function and its associated shape factors may depend on the receptor-analyte system, immobilization procedure, flow conditions and sensing platform.

### 2.2 Experimental Measurements

To assess the developed model, a commercial QCMD biosensor technology was employed to experimentally evaluate the interaction kinetics between a layer of surface-immobilized antibodies (anti-IgG) and its associated antigen (IgG) as an exemplar biological interaction. The mass deposited on the surface of a QCMD sensor can be estimated from the shift in resonant frequency, Δ*f*:

10 $$\Delta M=-C\times \frac{\Delta f}{n}$$

where *M* is the mass (*ng*), *C* is the mass sensitivity constant (*ng*/*cm*), *f* is the resonant frequency (*Hz*) and *n* is the harmonic used in the measurement. Hence, the mass deposited on the surface can be estimated for receptors as well as target biomolecules. To produce and analyze the model, two sets of QCMD data with different experimental procedure were used, both focusing on anti-IgG-IgG interactions. The first data set is based on our previously published study (see^23^ for experimental details), with a follow up set of experiments designed to specifically evaluate the influence of antigen concentration and flow rate on the binding kinetics.

For the flow rate experiments, QCMD measurements were performed on a QSense Pro (Biolin Scientific) using silicon dioxide coated sensors (QSX 303). QCMD sensors were cleaned by UV–ozone treatment (60 *min*, 100% power, 5 sccm O_2_), followed by sonication in a 2% Hellmanex III solution (Hellma Analytics, Müllheim, Germany) (10 *min*) and then sonication in ultrapure water (10 *min*). Cleaned sensors were dried with nitrogen gas then functionalized with polydopamine (15 *min*, 2 *mg*/*mL* dopamine in 5 *mM* Tris buffer) and loaded into the flow module. The temperature in the QCMD chamber was set to 21 °C (standard deviation <0.02°C/h) and flow rate was 20 *μL*/*min*, unless stated otherwise. All chemicals and solvents were used as supplied unless otherwise detailed. Dopamine HCl (≥98%), Tris buffer (premade pH 8.5 ± 0.2), phosphate-buffered saline (PBS) (preweighted tablets made up with Milli-Q grade water—pH 7.4 ± 0.2), goat anti-human IgG antibody—polyclonal, unconjugated, containing 0.2% sodium merthiolate as a preservative (Sigma product AP112 - “Highly purified human IgG isolated from pooled normal human serum”), and IgG from human serum—polyclonal, unconjugated, containing ≤1% sodium, no stabilizers (Sigma product I4506, ≥95%) were purchased from Sigma-Aldrich. SuperBlock was purchased from Thermo Fisher, and all solutions were prepared using Milli-Q grade water.

After immobilizing of receptors, three different concentrations of IgG, 100 *ng*/*mL*, 1 *μg*/*mL* and 10 *μg*/*mL*, were run over the surface for an hour each, with a 20 *min* wash using PBS between each step. Each experimental condition was independently repeated at least twice to assess experimental repeatability. Across the datasets reported here, the variation between replicate measurements was less than 5% consequently, two replicate measurements were retained for these conditions. The corresponding fitted kinetic parameters and their variability, reported as mean ± standard deviation (SD), are provided in the (Supporting Information Section S2). It is important to note here that, while it is possible to accurately estimate the concentration of IgG antibody-antigen complex, determining the number of available binding sites is challenging. In theory, every anti-IgG can have 2 binding sites, though as sites are non-homogeneous, the number of sufficiently well-orientated, accessible and non-denatured sites will be much lower. Based on previous experiment performed using the same experimental procedure, this interaction ratio has been calculated to be between 0.3 to 0.5, (i.e., anti-IgG:IgG = 1:0.3–0.5). As an alternative to assuming a binding ratio to model kinetics, the saturation level at equilibrium can be considered for a range of target biomolecule concentrations, where the saturation level can be directly linked to the analyte concentration. In the present work, the saturation level is determined during the calculation of the binding kinetics.

## 3 Results and Discussion

We first demonstrate the limitations of the classical approach to accurately model experimental QCMD data for anti-IgG-IgG interactions, based on our previously published study.^23^ This classical model, based on the Langmuir isotherm, assumes constant and homogeneous binding kinetics and neglects the formation of a boundary layer at the sensor surface. Key variables are required to build the system computationally and correctly apply the Langmuir isotherm with a mass transfer model. This includes the kinetic constant rates (*k*_*on*_ and *k*_*off*_) and the number of available binding sites. Figure 3 shows the predicted sensor response compared to the experimental measurements for anti-IgG-IgG interactions, for various *k*_*D*_ values and estimated final saturation levels, *θ̂*_*max*_ %. Figure 3 a exhibits the estimation of QCMD responses for anti-IgG-IgG interactions with *k*_*D*_ in a range of 1 *nM* to 4 *nM*, considering a fixed saturation level of 98%. While the best fit found for *k*_*D*_ is 2 *nM*, a clear discrepancy can be seen between the model and measurements for IgG concentrations greater than 10 *μg*/*mL*. There is significant overestimation in both equilibrium position and the rate in which equilibrium is reached. This can be observed for all *k*_*D*_ values, indicating the change of kinetic rates in higher concentrations. Further to that, the effect of saturation level was studied, where the kinetic constant of 4 *nM* (with *k*_*on*_ and *k*_*off*_ of 1.1 × 10^5^ (*M* ^–1^ *s* ^–1^) and 4.4 × 10^–4^ (*s* ^–1^) , respectively) is considered for binding sites in a saturation level of 95% to 100% (Figure 3 b). Although reducing the saturation level in the model improves the estimation of the sensor response for an analyte concentration of less than 1 *μg*/*mL*, the difference between the model and experiment increases drastically when analyte concentration is ≥10 *μg*/*mL* similarly to *k*_*D*_. In effect, no variation of model parameters in the classical model can accurately simulate the response observed in this experiment.

**Figure 3:**
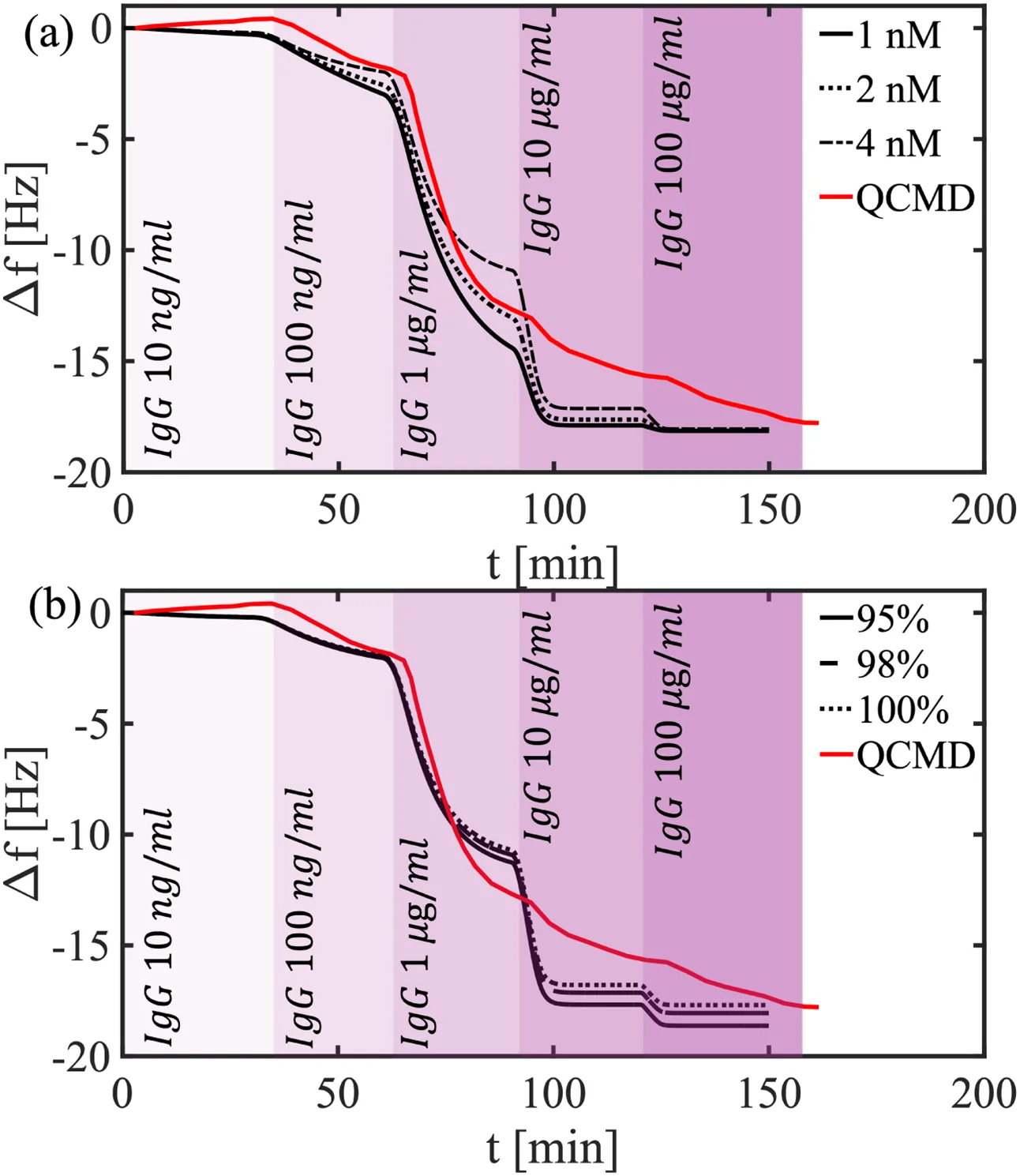
QCMD response (data from ref 23) predicted through considering the classical Langmuir model for (a) saturation (*θ̂_max_*) of 98% with *k_D_* in a range of 1 *nM* to 4 *nM* and (b) a *k_D_* of 4 *nM* with *θ̂_max_* of 95% to 100%.

The results presented in Figure 3 demonstrate the limitations of the classical approach to model microfluidic integrated biosensors. This is mainly due to the assumptions that the binding kinetics are homogeneous and dissociated analytes diffuse away from the sensor surface immediately. Here in Figure 3, it can be seen that the association rate at higher concentrations is more than its value at lower concentrations, between 10 *ng*/*mL* to 1 *μg*/*mL*. This can be linked to effects such as availability of binding sites and changes in the local surface environment during binding interaction, as discussed in previously studies.^16,18,19^ To identify and quantify such changes in the binding interaction as a continuous variation of kinetics, we analyzed the same experimental QCMD data of anti-IgG interactions by employing the proposed model presented in Figure 2. As can be seen in Figure 4) a, our new algorithm provides a more accurate model of the experimental observations for the anti-IgG-IgG interaction for concentration range of 1 *μg*/*mL* to 100 *μg*/*mL* IgG than the classical method. The error analysis is shown in Figure 4 c–d, demonstrating a consistent model prediction across a wide range of analyte concentrations. Here, the error metrics are reported only for IgG concentrations greater than 1 *μg*/*mL*. At lower concentrations, the QCMD response approaches the measurement noise floor, resulting in a reduced signal-to-noise ratio and increased uncertainty in the extracted kinetic parameters. Consequently, fitting errors at concentrations below 1 *μg*/*mL* are dominated by measurement noise rather than model performance and were therefore excluded from the quantitative error analysis in Figure 4. The proposed method shows an error less than 3% consistently with an NRMSE of 1.5%, in contrast to the classical Langmuir model that has errors from 5–30% if the classic binding kinetic exponential extraction method is utilized. This error for can be reduced by mathematically fitting the Langmuir equation, yielding an NRMSE of 5%, with a maximum error of 10%, occurring shortly post 10 *μg*/*mL* injection. Importantly, the proposed model predicts both the transient and steady-state binding responses for a wide range of IgG concentrations. The variation of the dissociation rate, *k*_*D*_, estimated using the proposed model reveals that *k*_*D*_ is constant (*k*_*D*_ = 4 *nM*) for up to 40% occupancy of the immobilized anti-IgG (Figure 4(b), which is the critical value *θ̂*_*c*_ in eqs 8 and 9. As can be seen, *k*_*D*_ subsequently reduces for binding interactions beyond 40%. The kinetic graph in Figure 4 b can be analyzed based on biosensor technology, biomolecular interactions and detection process. The developed kinetic models, eqs 8–9, fit for QCMD technology used to measure anti-IgG-IgG interactions, where *k*_*D*_ of 4 *nM* can be detected for homogeneous binding interactions. This dissociation rate constant has been reported through previous studies.^23^ Furthermore, results show *θ̂*_*c*_ = 40%, which can be linked to the testing procedure such as immobilization protocol, flow rate and the sensor surface characteristics. Hence the change of detection procedure can alter uniformity of receptors and binding interactions, considered here as *θ̂*_*c*_.

**Figure 4:**
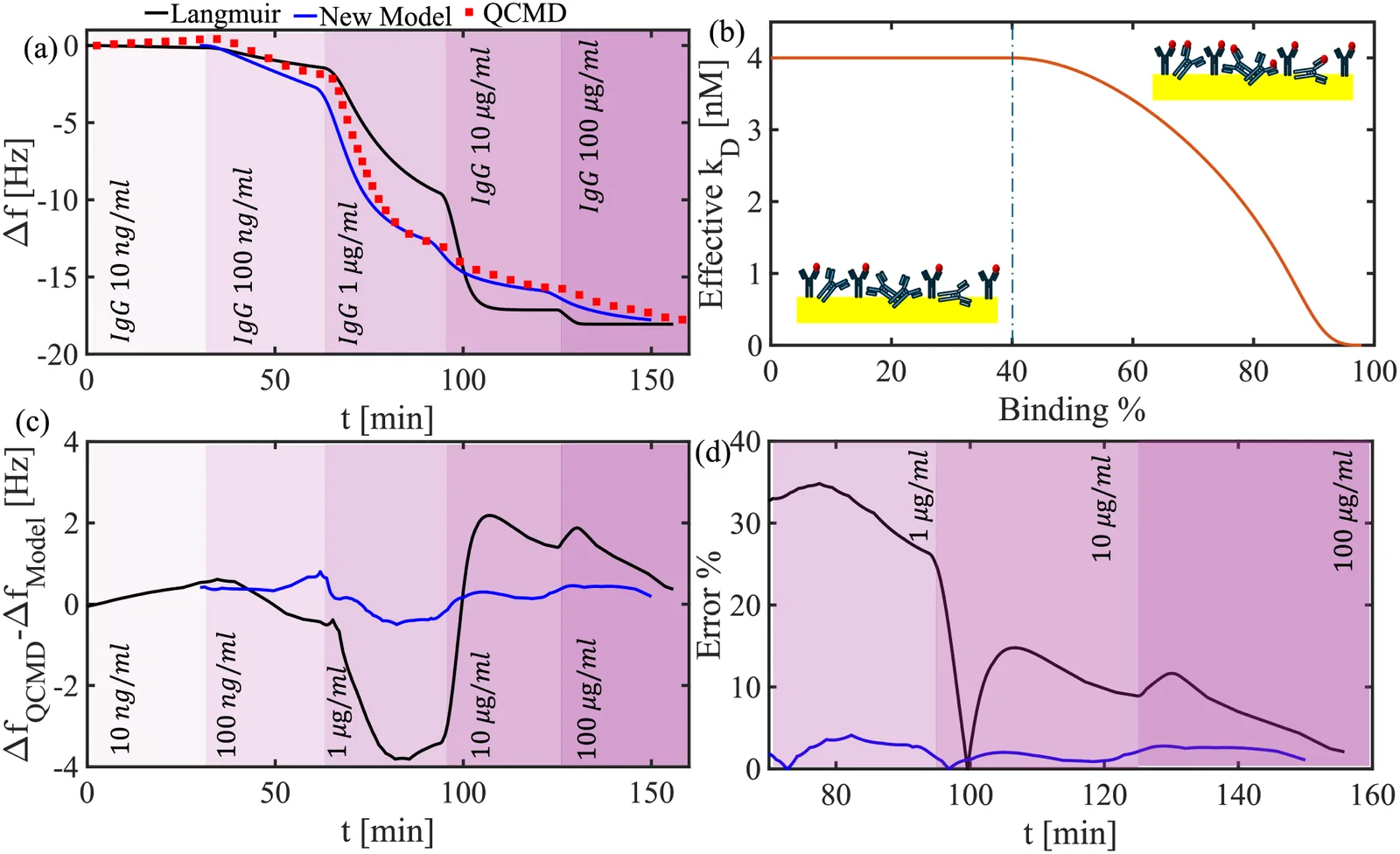
(a) QCMD response (data from ref 23) predicted through the proposed numerical approach and the classical Langmuir for anti-IgG-IgG interactions with IgG concentrations in the range 10 *ng*/*mL* to 100*μg*/*mL*. For the classic model the following kinetic constants were used, *k_on_* = 1.1 × 10^5^ *M* ^–1^ *s* ^–1^, *k_off_* = 4.4 × 10^–4^ *s* ^–1^, and *k_D_* = 4 *nM*. For the new model, the obtained optimum model constants are $k_{on}^{*}=1.8\times 10^{5}M^{-1}s^{-1}$ and $k_{off}^{*}=7.2\times 10^{-4}s^{-1}$ , and *θ̂_c_* of 40%, leading to (b) variation of effective *k_D_* as a function of binding level. The inset schematic shows how at *θ̂_c_*, most well-oriented and active binding sites are occupied as they would bind first, such that fewer active sites start to form binding complexes, reducing both the binding rate and the effective *k_D_*. Comparison between the classical Langmuir (black curve) and the new proposed model (blue curve) with experiments showed as (c) the difference in frequency and (d) error % for a range of IgG concentrations.

To further assess the performance of the proposed model against different mass transfer and immobilization conditions, a set of experimental measurements were performed again using QCMD to evaluate anti-IgG and IgG interactions but now at a range of flow rates from 10 *μL*/*min* to 40 *μL*/*min*. Details of these experimental measurements can be found in Section 2.2. These experiments allow us to directly evaluate the influence of flow rate and the boundary layer on the experimentally observed binding kinetics. Figure 5 shows the QCMD response predicted using our model. Here, the variation of binding kinetics is estimated on the basis of eqs 8 and 9 to find the best fit. The fitted values of $k_{on}^{*}$ and $k_{off}^{*}$ do not vary monotonically with flow rate, and these parameters should therefore not be interpreted as direct functions of flow alone. In addition to the change in hydrodynamic conditions, the experiments at different flow rates were performed as independent experiments. There will thus be subtle differences between experiments in the receptor density, orientation and accessibility, all of which will influence the apparent association and dissociation parameters. The fitted $k_{on}^{*}$ and $k_{off}^{*}$ therefore represent effective ensemble parameters that reflect both local surface properties and transport conditions. In contrast, *θ̂*_*c*_ exhibits an overall increase from approximately 41% to 50% with increasing flow rate, suggesting that enhanced analyte transport allows a larger fraction of the receptor population to contribute before the response departs from the approximately homogeneous kinetic regime. Because surface preparation and flow rate were not varied independently in this dataset, the present results should not be interpreted as establishing a unique causal relationship between flow rate and the fitted kinetic parameters. To assess the practical identifiability of the fitted parameters and evaluate the robustness of the model to parameter variation, sensitivity analyses were performed for $k_{on}^{*}$ , *θ̂*_*max*_ and *θ̂*_*c*_, with the results provided in the (Supporting Information Section S2). For flow rates of less than 40 *μL*/*min*, an error of less than 2% is observed between the proposed model and experimental measurements, while an error of approximately 3% is obtained for the flow rate of 40 *μL*/*min*. Using the classical method leads to error of ≈5% for flow rates less than 40 *μL*/*min*, while error of around 7% is obtained for flow rate of 40 *μL*/*min*, mostly impacting during the transient state.

**Figure 5:**
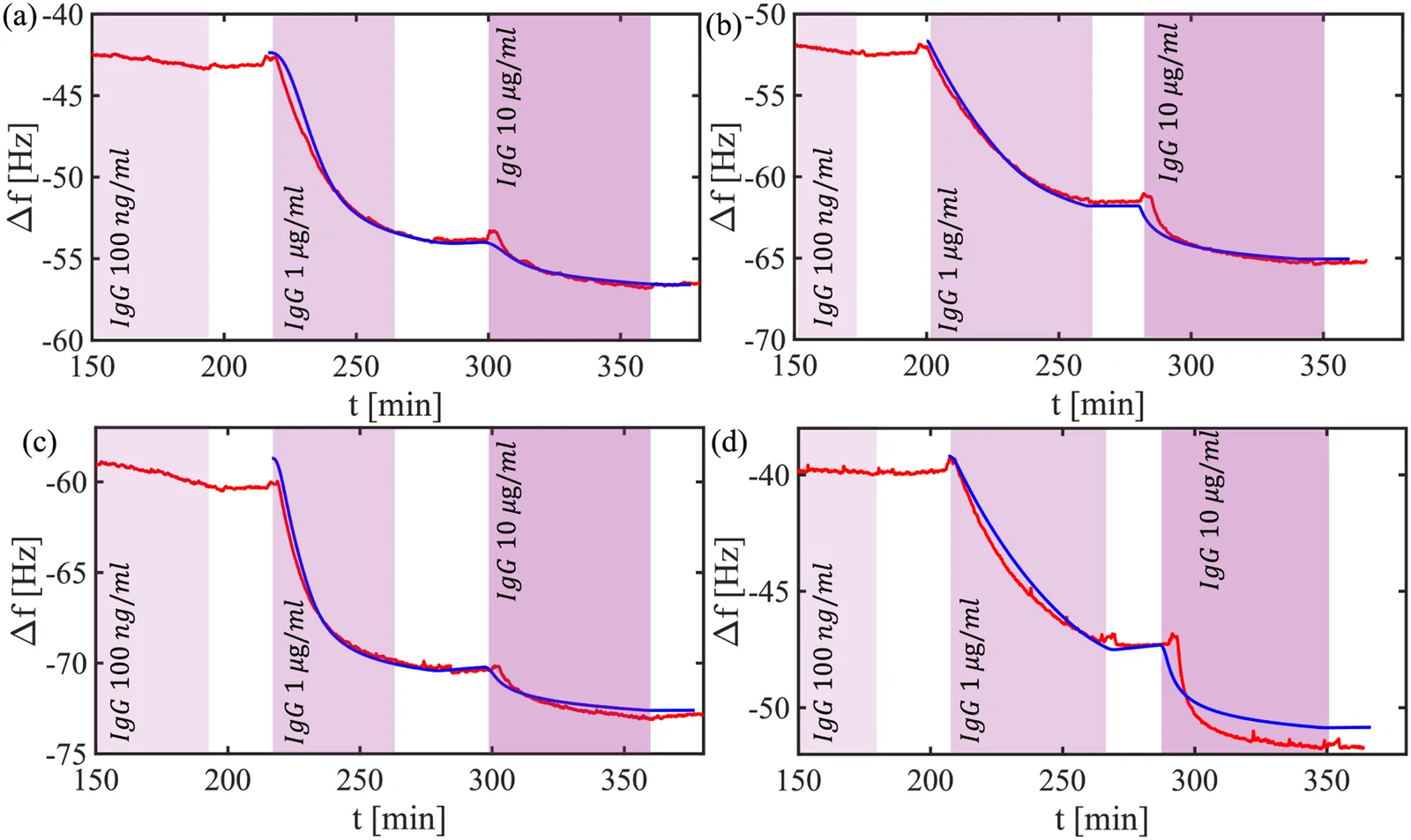
Measured QCMD for IgG antibody-antigen interactions and associated computational model (blue) for different flow rates of (a) 10 *μL*/*min* (model parameters of $k_{on}^{*}=1.1\times 10^{5}M^{-1}s^{-1}$ , $k_{off}^{*}=4.4\times 10^{-4}s^{-1}$ , *θ̂_c_* = 41%), (b) 20 *μL*/*min* (model parameters of $k_{on}^{*}=0.55\times 10^{5}M^{-1}s^{-1}$ , $k_{off}^{*}=2.2\times 10^{-4}s^{-1}$ , *θ̂_c_* = 43%), (c) 30 *μL*/*min* (model parameters of $k_{on}^{*}=1.4\times 10^{5}M^{-1}s^{-1}$ , $k_{off}^{*}=5.6\times 10^{-4}s^{-1}$ , *θ̂_c_* = 45%), and (d) 40 *μL*/*min* (model parameters of $k_{on}^{*}=0.45\times 10^{5}M^{-1}s^{-1}$ , $k_{off}^{*}=1.8\times 10^{-4}s^{-1}$ , *θ̂_c_* = 50%), for a range of the IgG concentrations of 100 *ng*/*mL*, 1 *μg*/*mL* and 10 *μg*/*mL*. Here, QCMD experiments run at 20 *μL*/*min* and 40 *μL*/*min* had a shorter (20 min) PBS wash step post superblock wash step compared to longer (40 min) PBS wash step for 10 *μL*/*min* and 30 *μL*/*min*.

Results in Figure 5 shows that increasing flow rate enhances the analyte flux and reduces the influence of local transport variability. Consequently, a broader population of receptor sites— including less accessible or less favorably oriented sites—contribute to the measured response. Because the observed kinetics become more representative of the overall receptor population, the range of surface coverage over which the apparent *k*_*D*_ remains approximately constant increases, leading to larger values of *θ̂*_*c*_. This behavior is consistent with established mass-transfer theory, which predicts that increasing flow rate reduces depletion effects and enhances analyte transport through the stagnation layer.^24,25^ Under these conditions, receptor populations with differing accessibility and orientation are sampled more uniformly, reducing the influence of local transport variability on the measured response. Consequently, a larger fraction of the binding process exhibits approximately constant *k*_*D*_, resulting in an increase in the critical coverage *θ̂*_*c*_. Finally, to assess whether the proposed kinetic framework is specific to the anti-IgG/IgG interaction, the model was further evaluated using an independent affinity interaction involving C-reactive protein (CRP) and its associated antibody immobilized on a QCMD sensor. The same modeling framework was applied without changing its underlying formulation, while the kinetic parameters were independently fitted to the CRP measurements. Tests with the new model again demonstrate improved fittings compared to the classical Langmuir, confirming the versatility of the model for describing a range of affinity interactions. Details can be found in the (Supporting Information Section S3).

Further supporting this concept, previous studies showed that mechanical forces modify both thermodynamic stability and kinetic rates by introducing a work term along the reaction coordinate. This alters the thermodynamics of the system including Gibb’s free energy, altering the apparent free energy of activation without changing the intrinsic molecular interaction pathway. Hydrodynamic flow introduces directional mechanical bias and reduced residence times.^26^ Consequently, variations in flow rate can modulate apparent activation energies and binding affinities by reshaping the energy states. Hence more binding interactions with the same apparent kinetics can dominate in higher flow rates, before less available sites begin to interact with analyte molecules.^27^ Recent advances in computational studies have highlighted that activation energies are strongly influenced by conformational sampling, non-covalent interactions, and dynamical effects rather than being fixed molecular constants, implying that experimental conditions that bias molecular trajectories, such as surface immobilization and flow, can modulate apparent kinetic barriers without altering intrinsic interaction energies.^28^ Beyond the flow rate,^29,30^ there are several additional experimental factors that influence the internal energy and binding kinetics, including solubility,^31^ electromagnetic alignment,^32–34^ temperature,^26,35^ and surface prefunctionalisation procedure.^7,36^ The work presented in this study focuses on the development of a new computational approach to measure, model and catalog these variations of binding kinetics in affinity biosensors, while explicit understanding of sensor characteristics and variation remains for further studies. For example, the proposed framework may also have potential application to QCM-D studies of binding interactions involving supported lipid bilayers (SLBs), including interactions of membrane-associated receptors with pathogens, toxins, or other biomolecular species.^37,38^ Such interfaces can exhibit heterogeneous and coverage-dependent apparent binding behavior, for which a single set of Langmuir kinetic parameters may provide an incomplete description. In principle, the present framework could be used to represent the progressive contribution of different binding environments through the coverage-dependent kinetic distribution while simultaneously accounting for near-surface transport and re-binding effects. This may provide an advantage for identifying transitions in apparent binding behavior that are not captured by a homogeneous single-site model. Application of the framework to SLB-based systems therefore represents an interesting direction for future experimental validation.

## 4 Conclusion

This study presents a novel computational framework for modeling binding kinetics in affinity biosensors by incorporating receptor heterogeneity and localized boundary layer effects. The proposed approach addresses some key limitations of the single interaction classical Langmuir isotherm, namely the assumptions of uniform binding site activity and constant binding kinetic parameters. Related approaches, such as AIDA, demonstrate important progress in this area and highlight the need to account for multiple surface interactions. However, representing these interactions using discrete kinetic parameters remains limiting. While introducing additional interaction types enables qualitative analysis with well-defined values for *k*_*D*_, *k*_*on*_ and *k*_*off*_, the large variance observed in these parameters prior to discretization indicates that significant underlying heterogeneity and complexity are lost in the simplification. Here we instead introduce an example normalized distribution to represent variable binding affinities and accounting for the boundary-layer re-binding of dissociated molecules. The proposed model improves the accuracy of biosensor response predictions while providing a phenomenological description of the evolution of apparent binding kinetics with increasing surface coverage and under varying analyte concentration and flow conditions. Experimental evaluation using QCMD measurements of IgG and CRP antibody–antigen interactions demonstrated improved model performance for the high-affinity interactions investigated here. The proposed framework should be regarded as a heuristic alternative to the homogeneous, constant-kinetics assumptions of the classical Langmuir model, particularly for experiments reaching sufficiently high surface coverage for heterogeneous binding behavior to become appreciable. The present validation is limited to two molecular interaction systems measured using QCMD, and the fitted parameters represent effective ensemble quantities rather than uniquely defined microscopic kinetic constants. Further evaluation using additional receptor–analyte systems, surface functionalization strategies, and transduction platforms will be required to establish the broader applicability of the framework and its potential utility for the design and optimization of affinity biosensors.

## Supplementary Material



## Notes

Numerical model implementation; numerical solver; boundary-layer treatment; error and sensitivity analyses for model fitting; experimental measurements and model fitting for C-reactive protein (CRP) interactions (PDF)
