# Supplementary material for: Characterizing Variations in Binding Kinetics of Affinity Biosensors: A Boundary-Layer and Site-Quality Approach
Source: ACS Omega. 2026 Sep 14;11(38):57935–44. doi: 10.1021/acsomega.6c07361 (PMC13629395; doi:10.1021/acsomega.6c07361)
Supplement: Supplementary file 1 [file ao6c07361_si_001.pdf]

Supporting Information

Characterising Variations of Binding Kinetics in Affinity Biosensors: A Boundary Layer and Site Quality Approach†

Brandon Tipper,‡ Lisa M. Miller,‡ Steven Johnson,‡,¶ and Mohammad Nasr Esfahani*,‡

‡*School of Physics, Engineering and Technology, University of York, York YO10 5DD, UK*

¶*York Biomedical Research Institute, University of York, York YO10 5DD, UK*

E-mail: mohammad.nasresfahani@york.ac.uk

S1 Numerical Model Implementation

S1.1 Numerical Solver

To implement the Navier-Stokes equations and concentration of analyte flowing a channel incorporated with the first-order Langmuir isotherm (presented in Eqs. (1)-(6)), model requires discretisation through a spacial grid. Hence, variables at different times and spatial positions can be noted in the form $X^n_{i,j}$,where:

- X is the variable that is considered (such as p for pressure, b for bindings.
- n is the time-step ($n + 1$ denotes next time-step)

†Submitted to ACS Omega

- i is the y spatial position ($i+1$ is y above, i–1 is y below)

- j is the x spatial position ($j+1$ is x to the right, j–1 is x to the left)

To iterate the previous continuous equations over time, they can be discretised onto a mesh using various difference schemes. For example, in the binding equation, $\frac{\delta\theta}{\delta t} \equiv \frac{\delta b}{\delta t}$ may be discretised as $\frac{\delta b}{\delta t} = \frac{(b_{i,j}^{n+1} - b_{i,j}^{n})}{dt}$ which is a forward time first order difference scheme. By discretising all of the terms and substituting them into Eq. 6, a discretised first order binding equation is created as:

$$b_{i,j}^{n+1} = b_{i,j}^{n} + \delta t * (k_{on} * c_{i,j}^{n} * (b_{max} - b_{i,j}^{n})) - \delta t * k_{off} * b_{i,j}^{n} \tag{S1}$$

With this Equation being used for the binding kinetics in a first-order program. The size of the mesh and appropriate values can be found through convergence testing, where variance in dx and dy were effectively arbitrary due to the heavily reaction limited system. Here the Perclet number is $> 1 \times 10^4$, with an effective depletion zone region of $\approx 20nM$. Additionally, time-step limitations follow from the Courant condition,

$$C = \frac{u\Delta t}{x} \leq C_{max} \tag{S2}$$

In this numerical solution, the largest time step is found through consideration of all rate changes throughout the simulation, ensuring values such as negative concentration do not occur. To model a 2D microfluidic channel, the following boundary conditions is considered:

Table S1: Numerical boundary condition to implement a 2D microfluidic channel with a sensor at the bottom of the channel.

Parameter	Interior	Inlet	Outlet	Walls	Sensor
u	eq. 2	$u = U0$	$\frac{du}{dx} = 0$ Zero Gradient	$u = 0$ No-Slip	$u = 0$
v	eq. 3	$v = 0$	$v = 0$	$v = 0$ No-Slip	$v = 0$
p	eq. 1	$v\frac{dp}{dx} = 0$	$p = 0$	$\frac{dp}{dy} = 0$	$\frac{dp}{dy} = 0$
c	eq. 5	$c = C0$	$\frac{dc}{dx} = 0$	$\frac{dc}{dy} = 0$ Homogeneous Neumann	$\frac{dc}{dy} = -\frac{1}{D}\frac{db}{dt}$ Neumann
b	N/A	N/A	N/A	N/A	eq. 6 (S1.1)

S1.2 Boundary Layer

In the proposed model here, a boundary layer is considered in the numerical simulations. Instead of dissociated molecules immediately diffusing back into the main fluid flow, the additional concentration is stored on the sensor surface itself, representing molecules quickly dissociating and rebinding. This concentration is considered to be a separate species, such that standard diffusion of concentration from the main flow is not impacted. This results in both the local concentration and the diffusive concentration being available for association in a given timestep - increasing binding rates without increasing association rate k_{on}, effectively giving a momentum / catalytic effect. To show the effect of such boundary layer in numerical solutions, the classical Langmuir model and the proposed approach are used to model QCMD measurements reported previously for anti-IgG-IgG interactions.[1] Implementation of the boundary layer is compared through numerical solutions without it (Figure S2). As can be seen in the figure, considering the boundary layer reduces the discrepancy between the model and experimental measurements, especially for IgG concentration less than $1\mu g/mL$. There is negligible impact of the boundary layer in the model for IgG concentration more than $10\mu g/mL$, due to the fast equilibrium and high concentration of analyte. The effect of the

boundary layer in the classical Langmuir model is more significant, where the normalised root mean square error (NRMSE) changes from 9.6% to 7.5% through including the boundary layer in the numerical solution. NRMSE equation included below.

$$\mathrm{NRMSE} = \frac{\sqrt{\frac{1}{N}\sum_{i=1}^{N}\left(y_i - \hat{y}_i\right)^2}}{\left(y_{max} - y_{min}\right)} \tag{S3}$$

where y_i is the experimental result, and $\hat{y}_i$ is the simulation result, with individual value at position i and total number of values N.

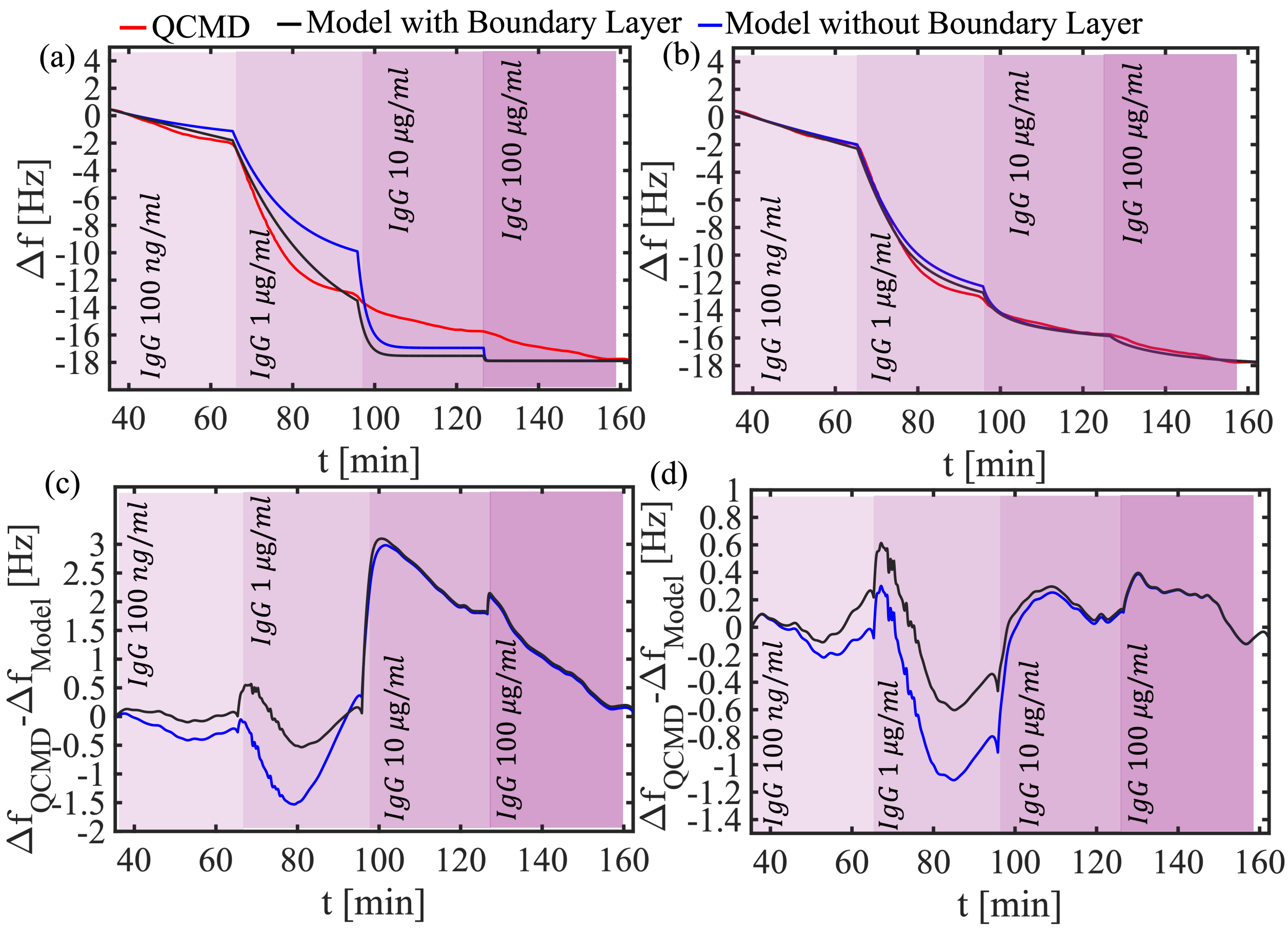

Figure S1: QCMD response (data from reference[1]) predicted through the (a) classical Langmuir model and (b) proposed numerical approach, while considering the boundary layer and excluding the boundary layer, compared with experimental QCMD measurements reported previously for anti-IgG-IgG interactions with IgG concentrations in the range 10 ng/ml to $100\mu g/ml$, leading to frequency difference between QCMD and model with and without the boundary layer presented for the (c) classical Langmuire and (d) proposed model.

S2 Error Analysis in Model Fitting

To demonstrate the reliability of the model, multiple simulations were consolidated and processed into the tables below. Specifically, k^*_{on} and $\hat{\theta}_{max}$ values were primarily determined using the initial rates of reaction, k^*_D was found using dissociation step washoff and the classical exponential fitting method, with $\hat{\theta}_c$ being found through manual variation to find the minimal NRMSE. Flow experiments (Figure 5) were run twice to find the standard deviation, with experiment[1] being averaged from multiple suitable modelling results.

Table S2: Fitted parameters in the proposed model and associated standard deviations to various QCMD measurements with estimated NRMSE.

QCMD Experiment	k^*_{on} $\times 10^5$ $[M^{-1}s^{-1}]$	k^*_D $[nM]$	$\hat{\theta}_c$ %	$\hat{\theta}_{max}$ %	NRMSE
Reported in[1]	1.385 ± 0.035	$2.5nM \pm 1.5$	44.5 ± 0.5	98 ± 1	1.455 ± 0.015
Figure 5 (a)	0.825 ± 0.025	$1.5nM \pm 0.5$	40.0 ± 1.0	90 ± 1.5	1.385 ± 0.105
Figure 5 (b)	0.525 ± 0.025	$1.5nM \pm 0.5$	41.0 ± 1.0	90 ± 1.5	1.815 ± 0.145
Figure 5 (c)	1.20 ± 0.05	$1.5nM \pm 0.5$	32.5 ± 2.5	90 ± 1.5	1.60 ± 0.17
Figure 5 (d)	0.48 ± 0.05	$6.5nM \pm 1.5$	15.0 ± 5	80 ± 5	3.06 ± 0.28

Table S3: Fitted parameters in the classical Langmuir and associated standard deviations to various QCMD measurements with estimated NRMSE.

QCMD Experiment	k^*_{on} $\times$ 10^5 $[M^{-1}s^{-1}]$	k^*_D $[nM]$	$\hat{\theta}_{max}$ %	NRMSE
Reported in[1]	1.75 ± 0.25	2.5 ± 1.5	99.25 ± 0.25	4.82 ± 1.22
Figure 5 (a)	0.70 ± 0.05	0.1 ± 0.05	99.25 ± 0.25	4.82 ± 1.22
Figure 5 (b)	0.50 ± 0.05	0.1 ± 0.05	99.25 ± 0.25	5.66 ± 1.41
Figure 5 (c)	1.05 ± 0.10	0.1 ± 0.05	99.25 ± 0.25	5.93 ± 1.87
Figure 5 (d)	0.45 ± 0.05	0.1 ± 0.05	99.25 ± 0.25	6.84 ± 3.28

S2.1 Sensitivity Analysis

When fitting the model to previously reported QCMD measurements,[1] identification of the impact from the dissociation is difficult due to the experiment procedure having no explicit wash out step, with association dominating the response at all stages. Further to that, the high affinity of anti-IgG-IgG interaction results in the model being mainly sensitive to factors affecting k_{on}. Shown below is sensitivity analysis of k^*_{on}, $\hat{\theta}_c$, $k\alpha$ and $\hat{\theta}_{max}$.

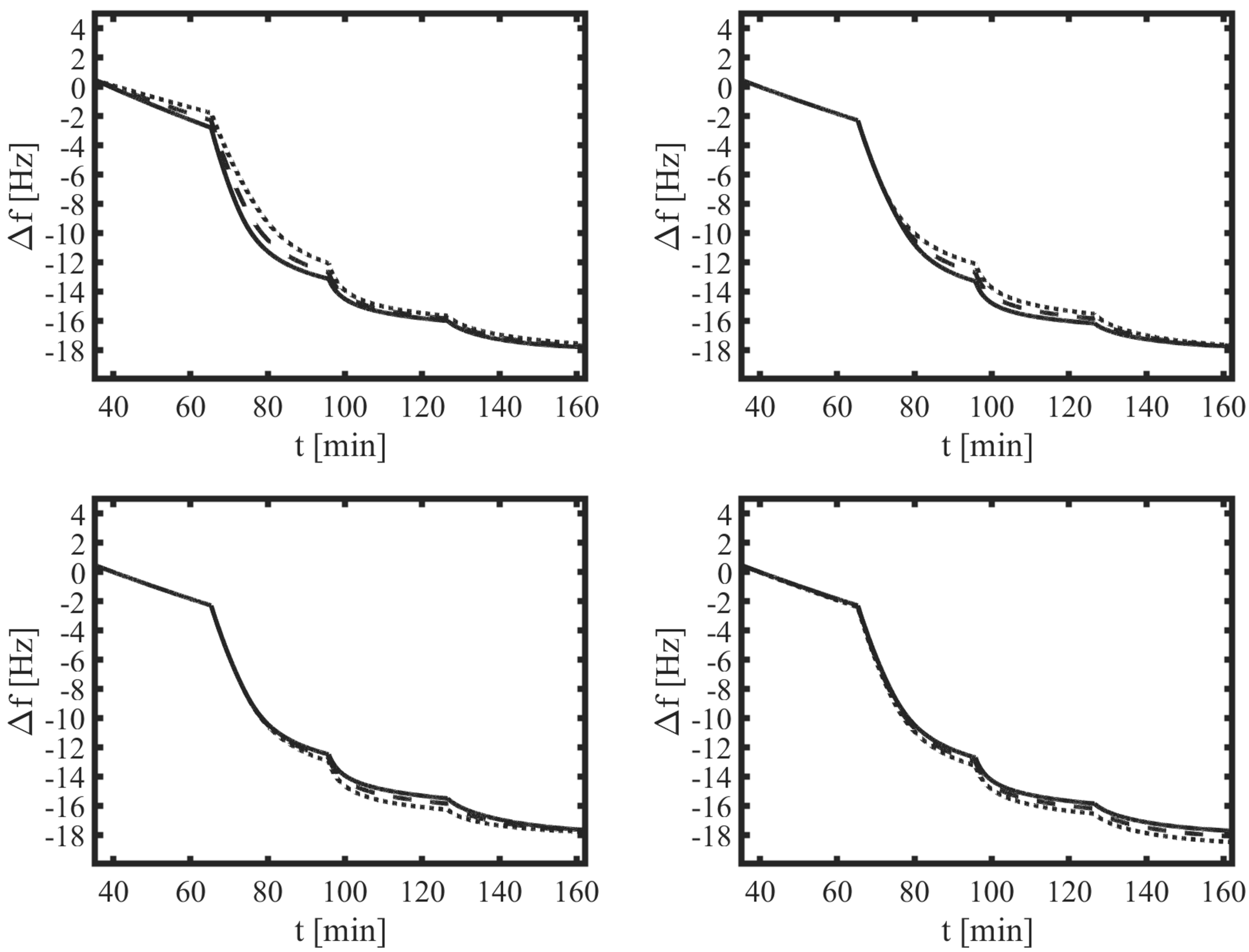

Figure S2: Sensitivity analysis of (a) k^*_{on}, (b) $\hat{\theta}_c$, (c) $k\alpha$ and (d) $\hat{\theta}_{max}$ for the model fitting shown in Figure 3. For (a) - (c), The dashed line follows the values reported in Table S2, with the dotted line having the respective value 20% lower and the solid line having a value 20% higher than the original value. For (d), The solid line has $\hat{\theta}_{max} = 99\%$, The dashed line has $\hat{\theta}_{max} = 97\%$ and the dotted line has $\hat{\theta}_{max} = 95\%$.

From the Figure, it is clear that k^*_{on} primarily effects the initial on rates for the first two simulated concentrations ($100ng/ml$ & $1\mu g/ml$), with $\hat{\theta}_c$ defining the sharpness of the second

and third concentration curves ($1\mu g/ml$ & $10\mu g/ml$). $k\alpha$ modifies the third and fourth concentrations ($10\mu g/ml$ & $100\mu g/ml$) by reducing the association rate more sharply, with $\hat{\theta}_{max}$ defining the final equilibrium position, scaling the response to fit within its boundary.

S3 Model Fitting to C-reactive protein (CRP) Interaction

S3.1 Experimental Measurements

The experimental documentation for the included section of a new CRP experiment is provided below. In the experiment, a concentration of 50 $\mu g/mL$ of CRP was run over a CRP antibody at $30\mu L/min$, with the experiment run twice to ensure reliability with an experimental NRMSE 2%. To test CRP interaction, QCMD measurements were performed on a QSense Pro (Biolin Scientific) using silicon dioxide coated sensors (QSX 303). QCMD sensors were cleaned by UV–ozone treatment (60 min, 100% power, 5 sccm O_2), followed by sonication in a 2% Hellmanex III solution (Hellma Analytics, Müllheim, Germany) (10 min) and then sonication in ultrapure water (10 min). Cleaned sensors were dried with nitrogen gas then functionalised with polydopamine (15 min, 2 mg/mL dopamine in 5 mM Tris buffer) and loaded into the flow module. The temperature in the QCMD chamber was set to 21 °C (standard deviation < 0.02°C/h) and flow rate was 30 $\mu L/min$, unless stated otherwise. All chemicals and solvents were used as supplied unless otherwise detailed. Dopamine HCl ($\geq$ 98%), Tris buffer (premade pH 8.5 +- 0.2), phosphate buffer saline (PBS) (preweighted tablets made up with Milli-Q grade water - pH 7.4 +/-0.2). The CRP antibody (CRP Monoclonal Antibody, Scripps laboratories) was diluted to 50 ug/mL in PBS. CRP isolated from human serum (Scripps laboratories) was diluted in PBS to the desired concentration. SuperBlock was purchased from Thermofisher, and all solutions were prepared using Milli-Q grade water.)

S3.2 Model Fitting

Figure S3 shows the predicted frequency change in the CRP binding interaction using the classical Langmuir and proposed approach compared with experimental measurements. Fitted parameters and error analysis are presented in table S4.

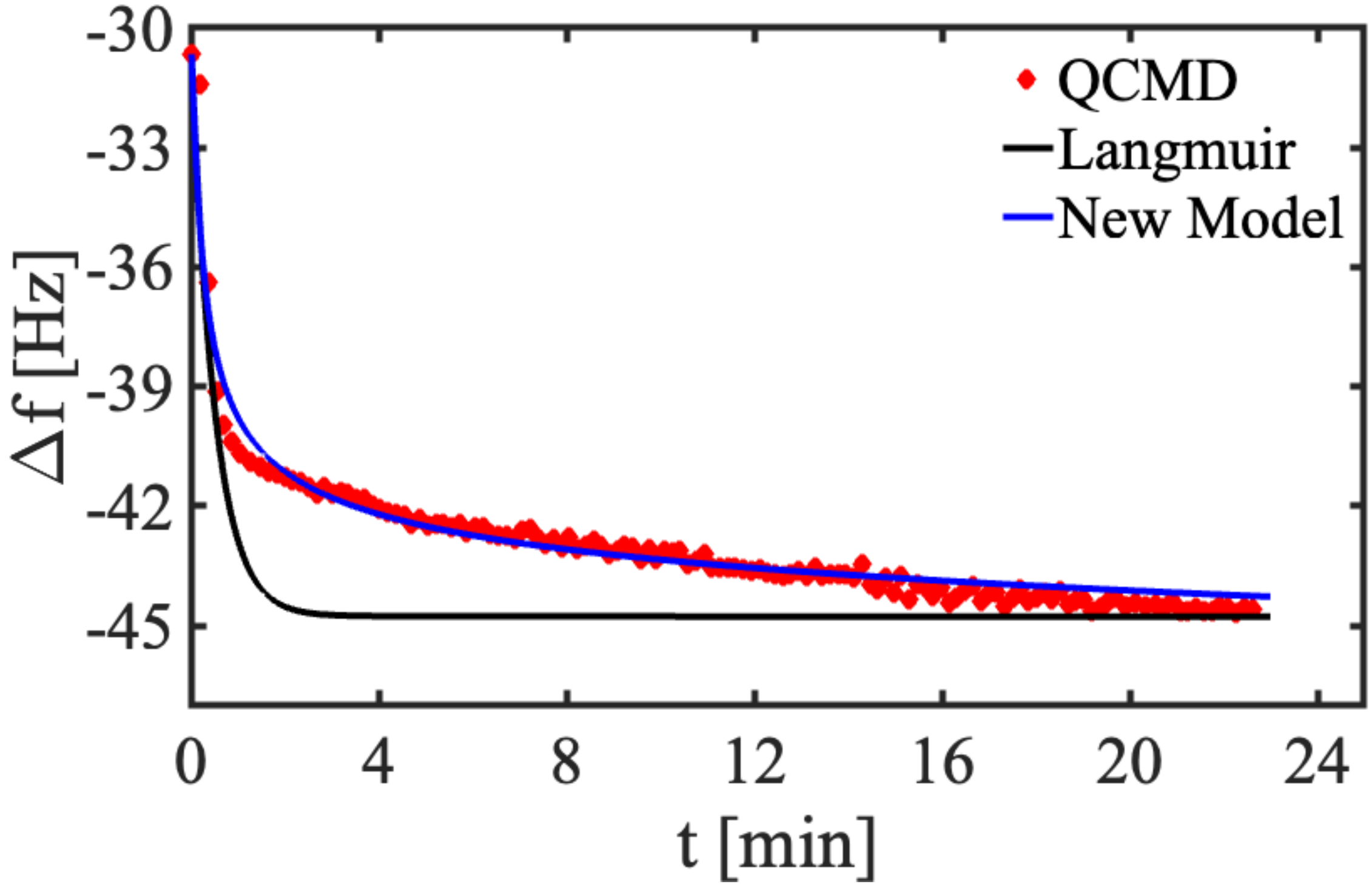

Figure S3: Frequency shift predicted through the classical Langmuir model and proposed model numerical approach compared with QCMD measurements for CRP binding interactions.

Table S4: Fitted parameters used for CRP interaction measurement with estimated NRMSE.

Model\Parameter	$k_{on} \times 10^5$ $[M^{-1}s^{-1}]$	$k_D[nM]$	$\hat{\theta}_c$	$\hat{\theta}_{max}$ %	NRMSE
Langmuir	0.75±0.10	2.5±2.0	N/A	99.25±0.25	8.20±1.01
Proposed Model	0.80±0.05	2.0±0.5	12.5±7.5	90±1.5	2.57±0.15

References

(1) Kenaan, A.; Li, K.; Barth, I.; Johnson, S.; Song, J.; Krauss, T. F. Guided mode resonance sensor for the parallel detection of multiple protein biomarkers in human urine with high sensitivity. *Biosensors and Bioelectronics* **2020**, *153*, 112047.
